# Functionally cured chronic hepatitis B: A cure‐related occult HBV infection state

**DOI:** 10.1002/mlf2.70102

**Published:** 2026-08-27

**Authors:** Fengmin Lu, Xin Zheng, Xinyu Du, Xianlin Ye, Yongzhen Liu, Jinfeng Zeng, Ying Yan

**Affiliations:** ^1^ Department of Microbiology and Infectious Disease Center, School of Basic Medical Sciences Peking University Health Science Center Beijing China; ^2^ Shenzhen Institute of Transfusion Medicine Shenzhen Blood Center Shenzhen China; ^3^ Department of Infectious Diseases The First Affiliated Hospital of Zhengzhou University Zhengzhou China; ^4^ MOE Key Laboratory of Model Animal for Disease Study, Model Animal Research Center, Department of Laboratory Medicine, Nanjing Drum Tower Hospital, Affiliated Hospital of Medical School Nanjing University Nanjing China; ^5^ National Center for Clinical Laboratories, Beijing Hospital, National Center for Gerontology; National Clinical Research Center for Gerontology; The Key Laboratory of Geriatrics of NHC; Institute of Geriatric Medicine Chinese Academy of Medical Sciences Beijing China

**Keywords:** chronic hepatitis B, functional cure, occult HBV infection

## Abstract

Occult HBV infection (OBI) is characterized by the presence of replication‐competent hepatitis B virus (HBV) DNA (covalently closed circular DNA [cccDNA] and/or relaxed circular DNA [rcDNA]) in the liver and/or HBV DNA in the blood, despite negative hepatitis B surface antigen (HBsAg) serology. The potential risks associated with OBI include HBV blood transmission, diagnostic challenges, and viral reactivation under immunosuppressive therapy. Some patients with OBI may face a higher risk of liver fibrosis compared to those who have achieved complete clearance of the virus. Functional cure is defined as the clearance of HBsAg, with or without hepatitis B surface antibody (anti‐HBs) seroconversion, and undetectable serum HBV DNA after finite therapy. It has been recommended as the optimal goal of antiviral treatment for chronic hepatitis B (CHB) by major guidelines. We highlight that patients achieving functional cure represent a distinct form of cure‐related OBI, with unique virological features and potential clinical implications. Furthermore, although direct epidemiological evidence remains limited, accumulating studies have demonstrated that OBI cases with undetectable HBV DNA and negative HBsAg serology retain the potential for transfusion transmission. Accordingly, the transfusion‐related risk associated with functional cure should not be overlooked. Here, we discuss the definition, virological and immunological basis, prevalence, diagnosis, and clinical significance of OBI, highlighting its link to cccDNA persistence and immune suppression of viral replication. Finally, we emphasize regular follow‐up to mitigate the risks of transfusion‐related transmission, HBV reactivation, and disease progression. Further research is warranted to elucidate the viral and immune status of functionally cured patients, thereby optimizing treatment strategies and ensuring blood safety.

## INTRODUCTION

Hepatitis B virus (HBV) infection remains a major public health challenge, with a 3.3% global prevalence and approximately 254 million chronic infections in 2022^1^. Following universal newborn vaccination since 1992, the overall serum hepatitis B surface antigen (HBsAg) positivity rate in China has decreased from 9.72% to 5.86% by 2020^2^. However, 75 million people still live with chronic HBV infection.

In 2013, Timothy M. Block and colleagues posed two critical questions:^1^ Is there a need for new drug categories to manage chronic hepatitis B (CHB)? and^2^ is a cure possible, and is it even necessary?^3^. They proposed two achievable types of cure. The first type is “functional cure,” in which patients resemble individuals who have achieved spontaneous clearance of HBV infection (anti‐hepatitis B core antibody [HBcAb] positive only), with outcomes equivalent to those with a normal expected lifespan. Another type is “apparent virological cure,” defined by the suppression or elimination of HBV viremia and antigenemia (collectively referred to as serologic viral markers) after discontinuing medication, along with the normalization of ALT (alanine aminotransferase) and other laboratory indicators. Since the equivalent “expected lifespan” associated with functional cure can only be ascertained through long‐term follow‐up, the authors proposed that virological cure is a more practical and measurable goal in both clinical practice and new drug trials.

In 2016, Sherman et al. acknowledged the difficulty of achieving a “true cure,” in which serological and virological markers, as well as intrahepatic covalently closed circular DNA (cccDNA) and integrated HBV DNA, are undetectable^4^. They then redefined the concept of “functional cure” as sustained undetectable levels of serum HBV DNA, hepatitis B e antigen (HBeAg), and HBsAg. Nevertheless, the possibility that ultra‐sensitive assays may still detect low levels of HBV DNA or antigens cannot be excluded.

As clinical practice evolves, the definition of functional cure in major guidelines has been continuously updated. For example, the Chinese guidelines evolved from the 2015 definition of “sustained virological response with HBsAg loss accompanied or not accompanied by hepatitis B surface antibody (anti‐HBs) appearance, normal ALT, and minimal or no hepatic histopathological lesions” to the 2019 definition of “constantly maintaining serum HBsAg negativity (with or without anti‐HBs appearance), undetectable HBV DNA, and normal liver biochemical indicators after treatment cessation.” This updated guideline adopted the concepts from EASL (2017)^7^ and AASLD (2018)^8^, and acknowledged that functional cure may not require the complete elimination of intrahepatic cccDNA. Consequently, patients achieving functional cure may harbor residual cccDNA despite undetectable serum HBV DNA and HBsAg. These individuals thus represent a distinct state of occult hepatitis B virus infection (OBI), which we term “cure‐related OBI.” Because cccDNA persists, these patients still face the risk of HBV reactivation and hepatocellular carcinoma (HCC)^5^, ^6^, ^7^, ^8^. Since 2019, the AASLD‐EASL HBV‐HDV Treatment Endpoints Conferences have recommended functional cure as the preferred primary endpoint for clinical trials evaluating new finite therapies^9^. China's 2022 guidelines emphasize pursuing clinical cure (functional cure) for suitable patients^10^. Thus, functional cure‐related OBI is an emerging entity.

## WHAT IS OBI?

OBI initially gained attention as a public health concern. Some recipients of whole blood, blood components, or blood products from HBsAg‐negative but HBcAb‐positive donors developed acute or chronic HBV infections^11^, ^12^. Investigations traced these transmissions to detectable HBV DNA in HBsAg‐negative donors. This highlighted the risk of HBV transmission from OBI carriers lacking HBsAg, making them ineligible to donate blood^13^. The 2018 Taormina workshop defined OBI as the persistence of replication‐competent HBV DNA (cccDNA) in the liver, occurring with or without detectable HBV DNA in the blood, in individuals who test negative for serum HBsAg using currently available assays^14^. We proposed that replication‐competent HBV DNA should include not only cccDNA but also relaxed circular DNA (rcDNA), given that cccDNA is the sole source of pregenomic RNA (pgRNA), and rcDNA is synthesized via reverse transcription within the nucleocapsid using pgRNA as a template^15^. Notably, rcDNA contains the full genome of HBV and can potentially be converted into cccDNA. Therefore, replication‐competent HBV DNA, defined by its capability of producing progeny virions and serving as the *bona‐fide* genetic basis of HBV reactivation, should also include rcDNA. Unfortunately, the 2018 Taormina workshop did not incorporate our suggestion to include rcDNA within the definition of replication‐competent HBV DNA^14^. The updated consensus categorizes OBI into two types: seropositive OBI, in which hepatitis B core antibody (anti‐HBc) and/or anti‐HBs are positive, and the relatively uncommon seronegative OBI, in which both anti‐HBc and anti‐HBs are negative, accounting for approximately 1%–20% of all OBI cases.

## THE OCCURRENCE OF OBI

Historically, most OBI cases originated either in asymptomatic individuals who had achieved spontaneous clearance of the virus after acute self‐limiting HBV infection, in those who had recovered from acute hepatitis B, or in individuals with chronic HBV infection who experienced spontaneous HBsAg seroclearance during long‐term interaction between HBV and the host immune response against viral infection. Currently, with more CHB patients achieving functional cure under optimized, precise, or even personalized therapy, cure‐related OBI is emerging.

### Virological and immunological basis

The natural history of chronic HBV infection involves continuous virus–host immune interactions^16^. Under sustained immune pressure, the infected hepatocytes are eliminated, and the overall cccDNA pool is significantly decreased^17^. Simultaneously, host epigenetic mechanisms silence residual cccDNA, allowing immune evasion^18^. However, this does not mean that cccDNA is completely transcriptionally silenced, even when the host's immune system effectively controls viral replication^19^.

### Virologic variation

**Figure 1:**
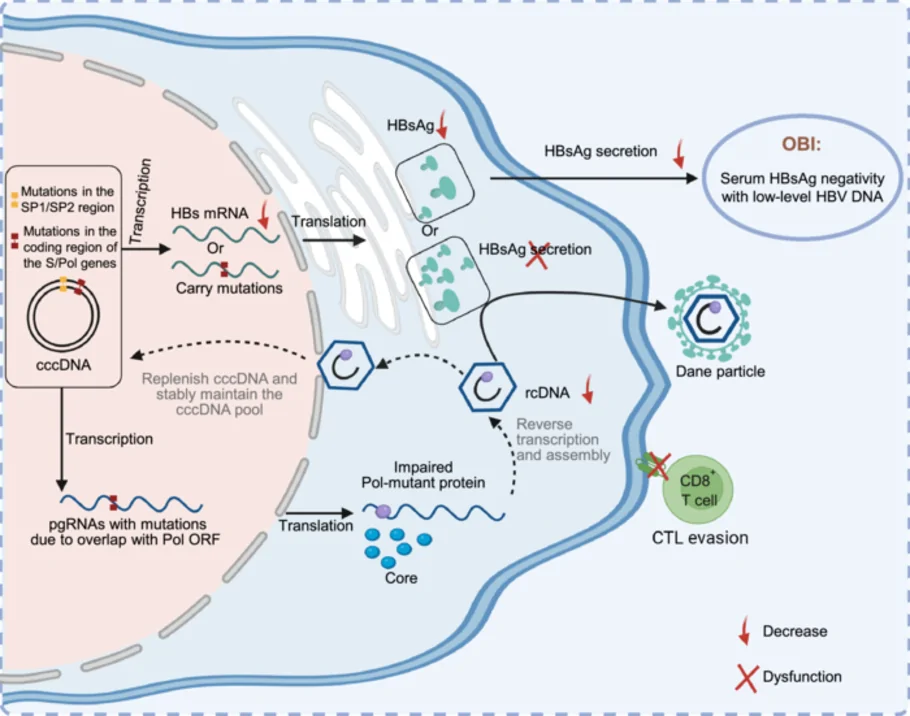
Virological and immunological basis for the occurrence of occult HBV infection (OBI). HBV mutations, on the one hand, lead to evasion of hepatitis B surface antigen (HBsAg) detection, reduced expression and secretion of surface antigen, or decreased viral replication, thereby reducing attacks by host cytotoxic T lymphocytes. On the other hand, some viral variations in the S gene result in reduced surface antigen production while retaining HBV polymerase protein function, which may facilitate the transfer of unsecreted HBV DNA into the nucleus, enhance replenishment of the cccDNA pool, and thereby maintain chronic HBV infection. cccDNA, covalently closed circular DNA; CTL, Cytotoxic T Lymphocyte; HBV, hepatitis B virus; pgRNA, pregenomic RNA; rcDNA, relaxed circular DNA.

The HBV genome, though only 3.2 kb in size, contains four promoters, two enhancers, four open reading frames (ORFs) encoding different viral proteins, and a series of regulatory elements^20^. This compact structure results in overlapping functional regions within the genome. Despite the constraints imposed by the genome's compactness, HBV undergoes a series of mutations to adapt to host immune pressure and maintain chronic HBV infection (Figure 1)^16^, ^18^. Some OBI cases involve mutations in the HBV S gene, leading to the production of HBsAg that is undetectable by current assays^21^. However, it is important to note that the S gene's ORF is entirely nested within the polymerase (Pol) ORF. Mutations in the major hydrophilic region (MHR) alter HBsAg antigenicity and impair virion secretion, potentially explaining the failure to detect HBsAg in individuals with OBI^21^. MHR mutations (e.g., G119R, C124Y, I126S, Q129R, S136P, C139R, T140I, K141E, D144A, and G145R) significantly impaired virion and/or S protein secretion in both Huh‐7 cells and mice. Mutations within the transmembrane domain of the S gene—such as C85R and F220C/Y—disrupt the transmembrane stability of HBsAg, thereby impairing its secretion^22^. Interestingly, mutations in the S gene often affect the reverse transcription/polymerase functions of the Pol protein. We recently found that some premature mutations in the S ORF, as well as in‐frame deletion mutations within the Pol ORF overlapping the S promoter (corresponding to the spacer region of the Pol ORF), may retain relatively intact Pol protein function crucial for HBV replication^18^. Therefore, it is reasonable to postulate that such mutations, which, on the one hand, retain Pol protein function and, on the other hand, evasion of host somatic HBsAg‑specific cytotoxic T‑cell clearance plays a pivotal role in the formation of OBI. The study also suggested that the lack of HBsAg, the envelope protein required for virion egress, may favor the maintenance of chronic HBV infection by enhancing intracellular replenishment of the cccDNA pool at a trace level. This is consistent with the occasional detection of low levels of HBV DNA, a characteristic feature of OBI, in the blood of some individuals with OBI^18^. Additionally, mutations such as T68I, S78N, and N98T in the pre‐S region have been shown to reduce HBsAg synthesis by inhibiting the activity of the pre‐S2/S promoter (Figure 1)^23^. The W62R mutation in the core gene reduced the production of hepatitis B core antigen (HBcAg) and HBeAg during HBV replication *in vivo*, potentially contributing to the occurrence of OBI^24^. Since the entire S gene overlaps the Pol ORF, these mutations invariably affect Pol function. Consequently, circulating HBV DNA levels in individual with OBI are usually low (<200 IU/ml), if detectable. In addition, the lack of normal L‐, M‐, and S‐HBsAg expression and efficient secretion would impair the release of Dane particles, which provides an alternative explanation for the low circulating HBV DNA levels observed in OBI^18^, ^25^.

## PREVALENCE OF OBI

Global OBI epidemiology varies, influenced by HBV prevalence and the sensitivity of detection assays. A systematic review of 305 studies showed that OBI prevalence parallels HBV endemicity among blood donors, being 0.06% (95% CI, 0–0.26%) in low‐endemicity countries, 0.12% (0.04%–0.23%) in intermediate‐endemicity countries, and 0.98% (0.44%–1.72%) in high‐endemicity regions^26^. Among HBsAg‐negative/anti‐HBc‐positive donors, well‐conducted studies reported circulating HBV DNA positivity ranging from 0 to 4.6%, with a median prevalence of 1%^27^. OBI occurs more frequently in individuals with co‐existing liver diseases such as hepatocellular carcinoma. As reported in a systematic review and meta‐analysis, the global OBI prevalence was 0.82% (95% CI, 0.69%–0.96%) in the general population, but increased to 13.99% (8.33%–20.79%) in patients with other liver diseases^28^. Similarly, in high‐risk groups, OBI prevalence was substantial regardless of HBV endemicity: 5.5% (2.9%–8.7%) in low‐endemicity countries, 5.2% (2.5%–8.6%) in intermediate‐endemicity countries, and 12.0% (3.4%–24.7%) in high‐endemicity countries^26^. Most studies focus on blood donors or patients with liver diseases, potentially limiting generalizability to the broader population.

OBI prevalence increases with age in individuals vaccinated at birth, presenting a new challenge for the prevention and control of OBI in the post‐universal infant vaccination era^29^. In China, for example, 57% of the population were anti‐HBc positive in 1992, prior to the implementation of universal hepatitis B vaccination for children, and higher anti‐HBc antibody titers may indicate an increased risk of OBI^30^, ^31^. As a consequence, most observed OBI cases showed isolated anti‐HBc positivity^32^. However, with the widespread implementation of neonatal hepatitis B vaccination, there has been a noticeable increase in the prevalence of anti‐HBs‐positive OBI in recent years^33^. On the one hand, infants born to HBsAg‐positive mothers who received active and passive immunization may remain OBI cases with anti‐HBs positivity. On the other hand, vaccine‐induced protection wanes over time. Consequently, these vaccine breakthrough asymptomatic infections gradually emerge with age, likely due to waning immune surveillance. In line with this, a recent report demonstrated transfusion‐transmitted HBV infection caused by blood characterized by positivity of anti‐HBs alone^34^. This shift highlights the evolving epidemiology of OBI in the post‐vaccination era, and the presence of OBI with sole anti‐HBs positivity among blood donors is an emerging issue.

## DIAGNOSIS OF OBI

It is not difficult to understand that the use of much more sensitive HBsAg testing kits can reduce false OBI, while the use of much powerful HBV DNA detection assays can also identify more OBI cases. For example, Caviglia et al. reported that 52% (52/100) of anti‐HBc‐positive liver donors met the criteria for status, which was defined by having positive nested‐PCR detection for at least two different HBV genomic regions. Also, of these, 52% (27/52) showed cccDNA positivity in liver tissues by droplet digital PCR, showing a median of 13 copies per one hundred thousand hepatocytes (95% CI: 5–25)^31^. While liver HBV cccDNA detection is most reliable, its invasiveness favors widespread blood HBV DNA testing. However, this approach may fail to detect intermittent low‐level viremia. Additionally, it has been reported that the minimum infectious dose required for HBV transmission is 0.15 IU/ml^12^, while the current limit of detection (LOD) for HBV DNA is typically 10–20 IU/ml in clinical practice​ and 1.4 IU/ml in blood screening.

Another important consideration is that anti‐HBc testing, though often used as a surrogate marker in epidemiology and donor screening, cannot serve as a definitive diagnostic indicator of OBI. This is because anti‐HBc is also frequently positive in individuals who have truly achieved clearance of HBV infection, meaning that there is no residual cccDNA in their liver tissue^35^.

Serum hepatitis B core‐related antigen (HBcrAg), which comprises three components, HBeAg, core antigen, and p22cr, is an emerging marker of chronic hepatitis B virus infection^36^. HBcrAg can remain detectable even when serum HBV DNA is undetectable or HBsAg loss has been achieved^36^. Since HBeAg and p22cr are translated from preC mRNA, whereas the core antigen is translated from pgRNA, and both viral RNAs can only be transcribed from cccDNA, we suggest that the diagnostic potential of serologic HBcrAg for OBI screening should be considered. Although HBV RNA levels may be elevated in patients receiving nucleos(t)ide analogues (NAs) antiviral therapy^37^, the current lack of international reference standards and traceability, along with the need for further validation of assay performance, including sensitivity, limits its use as a reliable predictor of OBI^38^.

## CLINICAL SIGNIFICANCE OF THREE DIFFERENT OBI STATES

**Figure 2:**
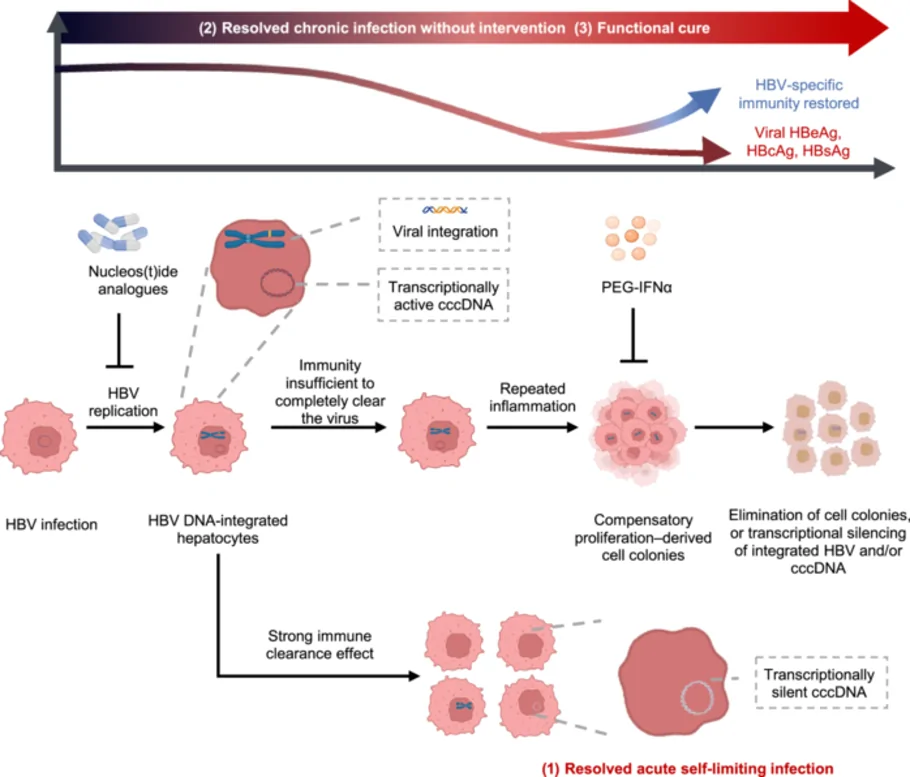
Distinct immunological and cellular outcomes of three types of OBI. (1) Resolved acute self‐limiting infection: Viral clearance occurs without repeated episodes of hepatic inflammation and hepatocyte death. (2) Resolved chronic infection without intervention and (3) functional cure: These outcomes often follow a chronic and long‐lasting “overt” HBV infection, where repeated cycles of hepatic inflammation and hepatocyte death promote compensatory proliferation of residual hepatocytes, particularly those harboring integrated HBV DNA, leading to the formation of clonally expanded cell colonies. In addition, while prolonged exposure to viral antigens induces progressive exhaustion of host HBV‐specific functional cellular immunity, the HBV‐specific immunity is thought to gradually recover in the later stages, as illustrated by the recovering trend line toward the resolved/cure states.

Seropositive OBI subjects may have lost HBsAg in three circumstances: following the resolution of acute HBV infection, through self‐healing after a chronic and long‐lasting “overt” HBV infection, and as a result of functional cure^14^, ^39^. Thus, the duration of serum HBsAg positivity before its disappearance may be highly variable^40^. The natural outcomes of acute and chronic HBV infection, as well as the outcomes of chronic hepatitis B under antiviral therapy, are illustrated in Figure 2. The types of OBI states and relevant characteristics are summarized in Table 1.

### OBI following the resolution of acute HBV infection

In general, exhaustion of host HBV‐specific cellular immune function is not expected to occur in individuals with OBI who have recovered from acute hepatitis B and/or self‐limiting acute infection, due to the absence of long‐term viral antigen exposure at high levels. Additionally, since the process of acute self‐limiting infection does not involve long‐term hepatic inflammation and repeated cell death and compensatory proliferation of residual hepatocytes, clonal proliferation should be rare. Undoubtedly, the risk of liver cancer should be the lowest among the three types of OBI. In line with this, OBI identified in blood donors rarely causes immediate clinical issues beyond reactivation and/or transmission risk^14^.

### OBI through self‐healing after a chronic and long‐lasting “overt” HBV infection

In this type of OBI, the risk of HCC may vary depending on the age at HBsAg clearance and the presence of liver fibrosis or cirrhosis. CHB progression is driven by virus–host interactions. Recent studies also suggest that OBI individuals may have a higher risk of liver fibrosis compared with those who have achieved spontaneous and complete clearance of previous HBV infection^41^. However, it remains unclear whether the severity of age‐related fibrosis/cirrhosis contributes to the risk of HCC even in individuals with spontaneous serum HBsAg seroclearance, or after antiviral therapy. Obviously, in individuals with OBI, especially those who spontaneously lost HBsAg during the natural history of chronic infection, a condition termed self‐resolved or OBI, the risk of HCC is strongly influenced by the age at HBsAg loss and fibrotic status^40^. Loss of HBsAg confers a lower risk of HCC, particularly when achieved before the age of 50^42^, ^43^, ^44^. Indeed, accumulating follow‐up studies in recent years have revealed that the risk of HCC may not be significantly decreased in individuals who lost serum HBsAg after the age of 50 years or in those with a cirrhotic background^45^, ^46^. These factors highlight the importance of careful monitoring and management of OBI in clinical practice. According to the EASL guideline, risk factors such as cirrhosis, metabolic liver disease, or coinfections can increase the estimated annual HCC risk in OBI individuals to >0.2% per year, and therefore, routine HCC surveillance is recommended for this population^47^. Similarly, the Chinese guidelines recommend that high‐risk patients undergo HCC surveillance at intervals of 3–6 months^10^. Unfortunately, the current World Health Organization (WHO) Hepatitis Elimination Strategy has ignored the diagnosis of OBI, even though there is growing evidence that links OBI to serious chronic hepatic complications, especially HCC^48^.

### OBI following functional cure (cure‐related OBI)

Another important category of OBI occurs in patients who have achieved functional cure. Although serum HBsAg and HBV DNA are undetectable, residual intrahepatic cccDNA may persist, representing a distinct cure‐related OBI state. As pointed out by Raimondo et al., it remains unclear whether patients achieving HBsAg seroclearance after antiviral treatment are comparable to those who have achieved spontaneous clearance of HBsAg, or whether the clinical implications are the same^14^. Potential persistence of intrahepatic cccDNA makes HBV reactivation and hepatitis, key clinical concerns during immunosuppressive therapy or chemotherapy. For example, among HBsAg‐negative and anti‐HBc‐positive patients with B‐cell non‐Hodgkin lymphoma, the proportion of HBV reactivation during B‐cell depletion therapy exceeds 9%^49^. Therefore, in functionally cured patients, a comprehensive evaluation of both the cccDNA pool size and residual integrated HBV DNA in the liver, along with their transcriptional activity, is critical. Additionally, the restored functional status of host HBV‐specific immune responses must be considered, as these factors collectively determine the risk of HBV reactivation and clinical outcomes^39^. Guidelines mandate routine screening for HBsAg, anti‐HBc, and/or HBV DNA for all patients starting immunosuppressive therapy^10^, ^47^. HBsAg‐negative, anti‐HBc‐positive, HBV DNA‐negative individuals should receive prophylactic nucleot(s)ide analogue therapy if they are scheduled to undergo immunosuppressive regimens associated with a high risk of HBV reactivation^47^. In particular, antiviral therapy is recommended for HBsAg‐negative and anti‐HBc‐positive patients who receive B‐cell‐targeted monoclonal antibodies, undergo hematopoietic stem cell transplantation, or have advanced liver fibrosis or cirrhosis^10^. These considerations underscore the importance of tailored long‐term monitoring and management for patients in the functional cure‐related OBI state. The types of OBI states and relevant characteristics are summarized in Table 1.

**Table 1:** Types of occult HBV infection (OBI) and their relevant characteristics.

	Plasma							
Type	HBcAb	HBV DNA^b^	Liver rcDNA/cccDNA	Antiviral therapy^c^	Risk of liver cirrhosis	Risk of hepatocellular carcinoma	Risk of blood or organ transplant transmission^d^	Risk of HBV reactivation
Resolved acute self‐limiting infection^a^	+(−)	—	+	>30 years of age	Extremely low	Extremely low	√ (if HBV DNA is detectable in blood)	√
Resolved chronic infection without intervention	+	—	+	Patient with liver cirrhosis	Significantly reduced, might be partially reversible	Significantly decreased, but may still be at higher risk if with cirrhosis, or at age >50 years with HBsAg loss	√	√
Functional cure	+	—	+	Patient with liver cirrhosis			√	√

^a^May present as acute hepatitis B or as an asymptomatic self‐limiting infection. ^b^For patients with chronic infection, if serum HBsAg is negative but circulating HBV DNA is detectable, the patient has not self‐recovered or achieved a cure. The possibility that ultra‐sensitive assays may still detect positive HBV DNA cannot be excluded. ^c^Following the guidelines for the prevention and treatment of chronic hepatitis B^10^. ^d^OBI in donor liver poses a risk of transmitting HBV to the recipient. ccDNA, covalently closed circular DNA; HBsAg, hepatitis B surface antigen; HBcAb, hepatitis B core antibody; rcDNA, relaxed circular DNA.

## CONCLUDING REMARKS

Functional cure, as defined by current guidelines, may place some patients in a cure‐related OBI state. This underscores the importance of regular surveillance after the disappearance of serum HBsAg. Although HBsAg and HBV DNA are undetectable, from a transfusion safety perspective, this emerging source of OBI warrants attention for potential transfusion‐related risk. It also highlights that such patients remain at risk of HBV reactivation, which should be carefully considered when immunosuppressive therapy is administered.
